# Physicochemical optimization of zinc oxide nanoparticles enhances their antimicrobial and anticancer activities via *RmpA*,* fnbA*, *cna*, and *LuxS* gene expression suppression

**DOI:** 10.1038/s41598-026-42733-3

**Published:** 2026-04-02

**Authors:** Mohamed khedr, Ahmed N. Emam, Mohamed Soliman Dora, Yasein Fadel Awadalla, Mostafa M. Al-Banna, Abdullah M. Nagib, Abdulrahman Hasib, Abdelrhman M. Abdelaziz, Loay A. Al-Dager, Amer Morsy Abdelaziz

**Affiliations:** 1 Botany and Microbiology Department, Faculty of Science, Al-Azhar University, Cairo, 11884 Egypt; 2 Refractories, Ceramics and Building Materials Department - Advanced Material Technology & Mineral Resources Research Institute, National Research Centre (NRC), El Bohouth St, Dokki, Cairo, 12622 Egypt; 3 Nanomedicine & Tissue Engineering Research Lab, Medical Research Centre of Excellence, National Research Centre, El Bohouth St, Dokki, Cairo, 12622 Egypt; 4 Faculty of Medicine, Tanta University, Tanta, Egypt

**Keywords:** ZnO-NPs, Virulence gene expression, Cytotoxicity, quorum sensing, *luxS, fnbA*, Biochemistry, Biotechnology, Cancer, Microbiology, Nanoscience and technology

## Abstract

Zinc oxide nanoparticles (ZnO-NPs) have gained attention for their anticancer and antimicrobial activity. Our study highlights a novel anti-virulence strategy against multidrug-resistant pathogens by showing that ZnO-NPs suppress bacterial virulence and quorum-sensing genes (*rmpA*,* fnbA*,* cna*, and *LuxS*) at sub-MIC levels. In this study, we synthesized ZnO-NPs using the chemical co-precipitation process, we confirmed their characteristics with the techniques TEM, XRD, UV-Vis spectroscopy, and measuring their zeta potential. ZnO-NPs are almost spherical, 30 nanometers in size, with a notable UV absorption at 375 nm and a zeta potential of -9.25 mV. ZnO-NPs showed impressive inhibition zones, especially against *E. coli*, with a zone size of 30.33 mm. The MIC of ZnO-NPs varied, with *Staphylococcus aureus* needing the highest concentration at 500 µg/mL, while *E. coli* and *Pseudomonas aeruginosa* needed 62.5 and 125 µg/mL, respectively. We also looked at how these particles affect cancer cells and found they reduced their growth in a dose-dependent way, with IC50 values of around 79 and 151 µg/mL for MCF-7 and HepG2 cells. Interestingly, when we examined the bacteria at the genetic level, we saw that ZnO-NPs at 62.5 µg/mL resulted in down-expression of key virulence genes like *rmpA*,* fnbA*, and *cna* to about 60% of normal levels, and the quorum-sensing gene *luxS* to 80%. This suggests that even at lower doses, the particles can weaken bacterial ability to cause disease without being fully bactericidal. Overall, our results emphasize how ZnO-NPs can be both antibacterial and anticancer agents, especially by targeting gene expression to boost their effectiveness.

## Introduction

Because multidrug-resistant bacteria (MDRB) can resist treatment by several classes of antibiotics, they pose a serious threat to global public health and can result in longer-lasting infections, higher mortality rates, and higher healthcare expenses^1^. Immunocompromised people and hospital environments are especially vulnerable to MDRB infections, which frequently lead to serious side effects like bloodstream infections, pneumonia, and surgical site infections^2^. Antibiotic overuse and abuse in agriculture and healthcare have sped up the emergence of resistance, decreasing the efficacy of existing therapies. As MDRB develop further, they present formidable obstacles to contemporary medicine, threatening advancements in critical care, chemotherapy, and surgery^3^.

Over the past ten years, ZnO-NPs have drawn a lot of attention because of their numerous uses in materials science, agriculture, and medicine. Of these, their strong antimicrobial and anticancer qualities hold particular promise for tackling contemporary issues like the emergence of bacterial infections that are MDRB and the restricted selectivity of conventional chemotherapy^4^. The biological activity of ZnO-NPs is attributed to their high surface area-to-volume ratio, enhanced reactivity, and capacity to produce reactive oxygen species (ROS)^5^. Chemically synthesized ZnO-NPs, particularly those made by co-precipitation techniques, have a number of benefits, such as ease of use, affordability, and controllable particle properties. This process usually entails the controlled heating of zinc acetate and sodium carbonate to create zinc carbonate, which is then calcined to produce ZnO-NPs^6^.

Prior research has shown that ZnO-NPs are effective against both Gram-positive and Gram-negative bacteria, frequently by rupturing the integrity of cell membranes, producing reactive oxygen species, and releasing Zn²⁺ ions that impede enzymatic activity^7^. Nevertheless, new data indicates that ZnO-NPs at sub-inhibitory concentrations may also reduce bacterial virulence by altering gene expression, a field that has not received enough attention. Particularly important for bacterial colonization, biofilm formation, and resistance are virulence factors like *RmpA*,* fnbA*,* cna*, and the quorum sensing gene *LuxS*. As such, they are desirable targets for alternative antimicrobial strategies. By focusing on these genes, it may be possible to reduce the pathogenicity of bacteria without applying significant selection pressure that encourages the emergence of resistance. ZnO-NPs have demonstrated potential in the treatment of cancer. ZnO-NPs cytotoxicity against different human cancer cell lines, such as liver (HepG2) and breast (MCF-7), is usually dose-dependent and associated with mitochondrial damage, oxidative stress, and the induction of apoptosis^8^. Comparative cytotoxicity between cell lines and specific mechanisms of selective anticancer activity, however, are still not well understood. The integrative analysis that links nanoparticle structure, colloidal properties, gene expression changes, and biological outcomes like bacterial inhibition and selective cytotoxicity is still lacking, despite significant advancements in our understanding of the physicochemical and biological effects of ZnO-NPs. The majority of current research ignores the molecular underpinnings of ZnO-NPs’ sub-MIC action, instead concentrating on the antimicrobial or anticancer effects separately. Furthermore, not many studies thoroughly assess how structural characteristics like particle size.

The novelty of our manuscript lies in demonstrating Gene-level suppression of bacterial virulence and quorum-sensing pathways, including RmpA, fnbA, cna, and LuxS. While earlier studies have focused mainly on the bactericidal or growth-inhibition properties of ZnO-NPs, our work uniquely highlights their ability to attenuate pathogenicity even at sub-MIC levels, which represents a promising anti-virulence strategy for combating multidrug-resistant pathogens. To close this gap, ZnO-NPs was synthesized using a chemical co-precipitation method, and their structural, morphological, and colloidal properties were characterized using zeta potential analysis, TEM, XRD, and UV-Vis spectroscopy. Additionally, we evaluate their cytotoxicity against MCF-7 and HepG2 cancer cell lines, ascertain MICs, and assess their antibacterial efficacy against clinical isolates. In order to maximize the therapeutic effect of ZnO-NPs and minimize the development of resistance, we explained the mechanism of ZnO-NPs as critically investigating the downregulation of important virulence genes at sub-inhibitory concentrations.

## Materials and methods

### Materials

Analytical-grade chemicals and solvents were all used without more purification. Zinc acetate dihydrate (Zn (CH_3_COO)_2_.2H_2_O, 99.99%) from Sharlab S.L. Chemicals in Spain. Sodium carbonate (Na_2_CO_3_, 98%) from EL-Gomhoria Chemicals in Egypt. Mueller–Hinton Agar (Oxoid Limited, Cat. No. CM0337). Antibiotic imipenem 10 was purchased from Oxoid Limited. SRPMI-1640 medium, MTT, and DMSO were obtained from Sigma Co., St. Louis, USA, and fetal bovine serum was from GIBCO, UK.

### Preparation of Zinc Oxide Nanoparticles

ZnO nanoparticles were synthesized by wet chemical precipitation methods with slight changes, adhering to previously known protocols^9,10^. 500 mL of 0.1 M sodium carbonate solution was incrementally introduced to an equal volume of 0.1 M zinc acetate dihydrate solution at 80 °C for two hours, with constant stirring of 700 rpm during the synthesis process to guarantee excellent particle formation kinetics. The pH of reaction was kept and adjusted after addition up to 6.5–7. Centrifugation was employed to isolate the resulting white solid, while residual starting ingredients were eliminated through repeated rinsing with distilled water. The purified precipitate was subjected to heat treatment at 300 °C for four hours under air conditions, after an overnight drying at 100 °C, The net yield the as-prepared final ZnO nanoparticles is about ~ 2.9–3 g.^11,12^.

### Characterization

Optical properties, such as the UV-Vis absorption spectrum have been recorded using TG-80 PG instruments, England, within the range 200–900 nm, with an increment 5 nm. The morphological properties were investigated using transmission electron microscopy (TEM/HR-TEM) JEM-2100 F, JEOL, Japan, operating at 160 KV. Furthermore, a Bruker D8 advanced X-ray powder diffractometer has been used to perform crystallographic property measurements on a Cu target with Kα1 = 1.54060 Å, Kα2 = 1.5444 Å, with 2θ step 4°, and recorded at a range of 10° to 70°. Additionally, using backscatter optics and a He/Ne laser (633 nm) at a 173° angle, the Malvern Zeta-sizer Nano ZS instrument measured the colloidal properties of the as-prepared nanoparticles, including particle size distribution and zeta potential^13^.

### Well diffusion assay

Nutrient Agar (NA) was used to test ZnO-NPs’ antimicrobial properties. NA plates were inoculated with six clinical isolates with concentration 10 ^6^ / ml of *Escherichia coli BAA-3286*,* Pseudomonas aeruginosa*,* Klebsiella pneumoniae*,* Staphylococcus aureus*,* Bacillus cereus*, and *Acentobacter* sp. 100 µl of ZnO-NPs (1000 µg/ml) were added to wells (6 mm), and the wells were pre-incubated for two hours at 4 °C. After the plates were incubated, the inhibition zones were measured. The antibiotic imipenem 10 was used as a positive control^14^. Experimental data were analyzed using one-way ANOVA, followed by LSD at 5% to compare means. All experiments were triplicated, and discriminant analysis was performed using SPSS (2009) at *p* < 0.05.

### MIC determination

Using the broth microdilution method outlined by ^15^, the minimum inhibitory concentration (MIC) of ZnO-NPs was assessed against clinical isolates of *Escherichia coli* BAA-3286, *Pseudomonas aeruginosa* Migula - BAA-2110, *Klebsiella pneumoniae* BAA-3267, *Staphylococcus aureus* 23,235-BSL 2, *Bacillus cereus* ATCC-14,579, and *Acentobacter baumannii* BAA-3339. ZnO-NPs were serially diluted in a 96-well microtiter plate by combining 100 µl of test sample with 100 µl of double-strength Mueller-Hinton broth. This allowed for the acquisition of final concentrations of 1000, 500, 250, 125, 62.5, 31.25, and 15.62 µg/ml. 50 µl of bacterial suspension, excluding negative controls, was added to each well after it was adjusted to the 0.5 McFarland standard. While sterile distilled water and broth were present in negative control wells, only broth and bacterial suspension were present in positive control wells. 30 µl of 0.02% resazurin was added following a 24-hour incubation period at 37 °C. Bacterial growth was indicated by a change in color to pink, red, or purple. Every experiment was run twice, and the mean MIC values were noted.

### Cytotoxicity Determination Using MTT Assay

ZnO-NPs’ anticancer properties were assessed against two cancer cells, Michigan Cancer Foundation-7 breast cancer (Mcf7) and the human hepatocellular carcinoma cell line G2 (HepG2), as follows: To enable the formation of a complete monolayer, 96-well tissue culture plates were seeded with 1 × 10⁵ cells/ml (100 µl per well) and incubated for 24 h at 37 °C. The growth medium was taken out once confluence was achieved, and wash media was used twice to clean the cell monolayer. The test sample was serially diluted twice in RPMI medium with 2% serum added. Three wells were kept as untreated controls, and each dilution (100 µl) was added to the designated wells. The cells were examined for indications of cytotoxicity, including loss of monolayer integrity, cell shrinkage, rounding, granulation, or detachment, following incubation at 37 °C. After adding 20 µl/well of MTT solution (5 mg/ml in PBS), the mixture was shaken at 150 rpm for five minutes. It was then incubated for four hours at 37 °C with 5% CO₂. After removing the medium, 200 µl of DMSO was used to dissolve the formazan crystals, and they were shaken to guarantee full solubilization. Optical density was correlated with cell viability, and absorbance was measured at 560 nm after subtracting background at 620 nm. To evaluate the cytotoxic effects on the cells, morphological changes were evaluated. Changes in cell shape, cytoskeletal structure, and membrane integrity were observed^16^.

### Gene expression

#### Total RNA isolation and cDNA synthesis

Applying ZnO NPs in concentration 62.5 µg/mL on the tested bacterial cells followed by incubation for overnight, then after centrifugation, cell pellets were suspended and initial homogenized in TRIzol reagent (Gibco) and freezing at -80 °C for at least half an hour to guarantee efficient cell lysis, total RNA was extracted from bacterial cells. The aqueous RNA-containing layer was meticulously collected and purified using RNeasy micro spin columns following phase separation using 1-bromo-3-chloropropane (Sigma Aldrich). A NanoDrop spectrophotometer (Thermo Fisher) was used to assess the isolated RNA’s concentration and purity, and gel electrophoresis on E-gel EX 2% agarose gels (Thermo Fisher) was used to confirm the integrity of the RNA^17^. The Advantage RT-for PCR Kit (Clontech, Palo Alto, CA, USA) was used to synthesize first-strand complementary DNA (cDNA). Using particular primers listed in Table 1, reverse transcription PCR (RT-PCR) reactions were carried out in 50 µL volumes. 75 ng of synthesized cDNA, 200 µM of each dNTP, 0.1 µM of each primer, 1.5 mM MgCl₂, and 1 unit of Taq DNA polymerase (Takara) were included in each reaction. Sterile distilled water was used to adjust the remaining volume. Initial denaturation at 95 °C for 4 min was followed by 33 cycles of denaturation at 95.6 °C for 1 min, annealing at 59.1 °C for 30 s, and extension at 72.5 °C for 90 s. At 72.5 °C, a last extension step was carried out^18^.

#### Real-time PCR amplification conditions

Complementary DNA (cDNA) from six samples four treated and two controls as well as particular primers indicated in Table 1 were used in semiquantitative PCR. Two QuantiTect SYBR^®^ Green RT Mixes (Fermentas) were used to conduct the reaction. 12.5 µl of SYBR mix, 1 µl of cDNA (50 ng), 1 µl of 25 pmol/µl of each forward and reverse primer, and 9.25 µl of RNase-free water were all included in each 25 µl reaction mixture. Samples were briefly centrifuged before being put into the rotor wells for loading. Initial denaturation at 94.7 °C for 90 s, 30 cycles of denaturation at 94.7 °C for 30 s, annealing at 57 °C for 60 s, and extension at 73.2 °C for 60 s were the PCR cycling conditions. The annealing and extension temperatures for the RecA gene were set at 50 °C and 69.7 °C, respectively. The Rotor-Gene Q 6000 system (Qiagen, USA) was used for amplification, and Ct values were noted^18^.

**Table 1:** specific primers for virulence factors and housekeeping gene.

primers	sequence 5 − 3	Tm (^o^C)	Product size	Ref
rampA-FW	AGGGAAATGGGGAGGGTACA	54	168bps	Our study
rampA-RW	GCAGCACTGCTTGTTCCTTT	56		
luxS-Fw	ATCCATACCCTGGAGCACCT	51.7	168bps	
luxS-RW	GCCACACTGGTAGACGTTCA	50.9		
fnbA-FW1	AGCGGTAACCAGTCATTCGAG	51	168bps	^19^
fnbA-RW1	TTGGCGGCGTTGTATCTTCT	51		
fnbA-FW2	GAAGATACAACGCCGCCAAC	50	212bps	
fnbA-RW2	AGGTTCTTCTTTTGCGGGTG	50		
fW 1RecA	GCCCTAATTGGTCCAGGCG	52	170bps	^20^
RW 1 recA	ACAACGGCGTTCTCTCCTAT	50		
FW-cna1	AAGGTGAACAGGTGGGTCAA	50	220bps	^19^
RW-cna1	CACTACTTGTTCCCGCTTCA	50		

## Results and discussion

### Synthesis and characterization of Zinc oxide nanoparticles

**Fig. 1:**
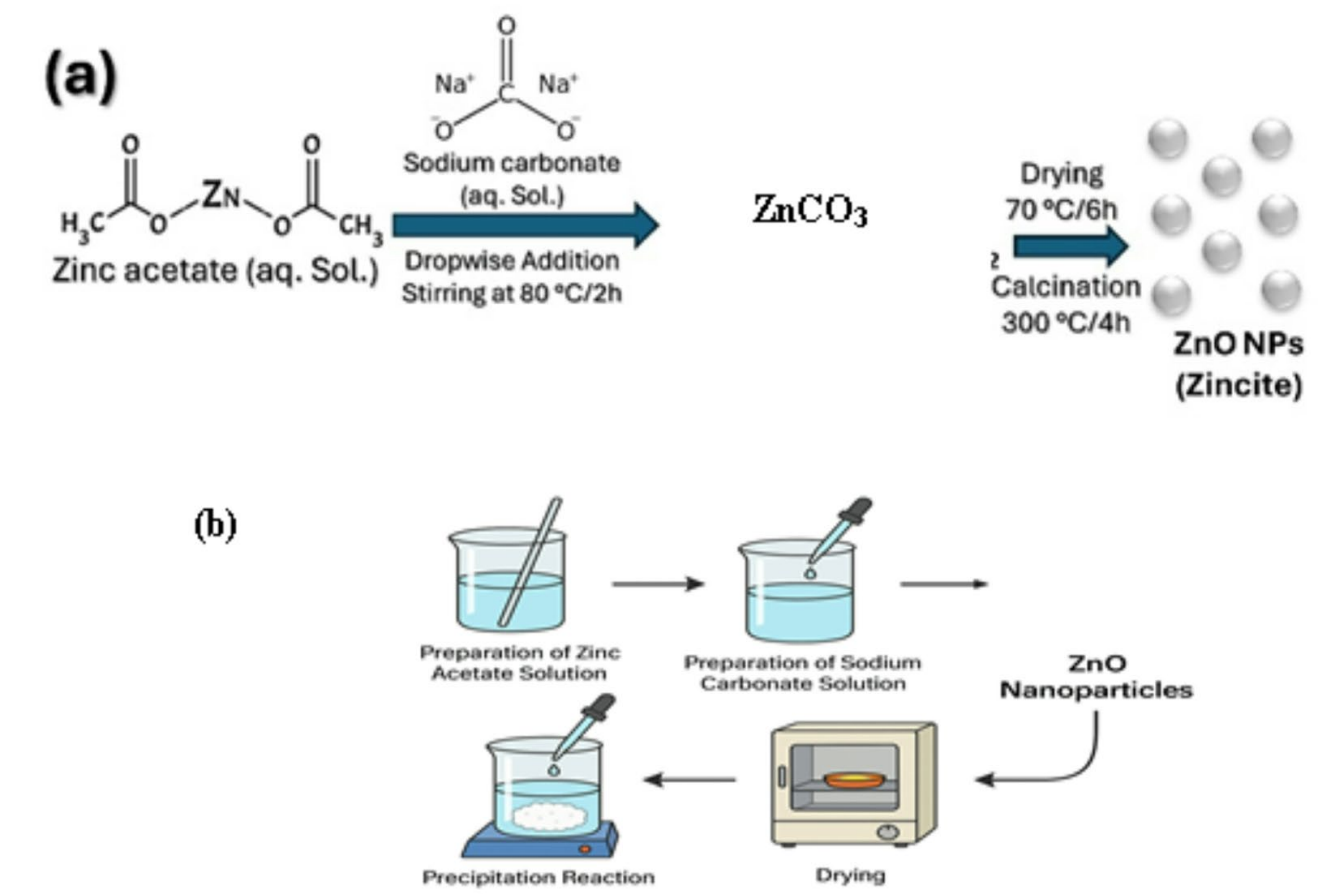
Schematic representation for fabrication of ZnO Nanoparticles.

The process for preparation of ZnO NPs via chemical co-precipitation method initiates with zinc acetate in an aqueous solution to which a sodium carbonate solution is slowly added while mixing at 80 °C for 2 h. By this reaction, an intermediate product called zinc carbonate (ZnCO_3_) is obtained. Then comes the drying part at 70 °C for 6 h, followed by the essential calcination phase at 300 °C for 4 h ^9,21,22^. During calcination, the zinc carbonate is converted to zinc oxide nanoparticles. The final product is shown to be minute round particles of ZnO NPs (Zincite) (See Fig. 1)^11,12^.

**Fig. 2:**
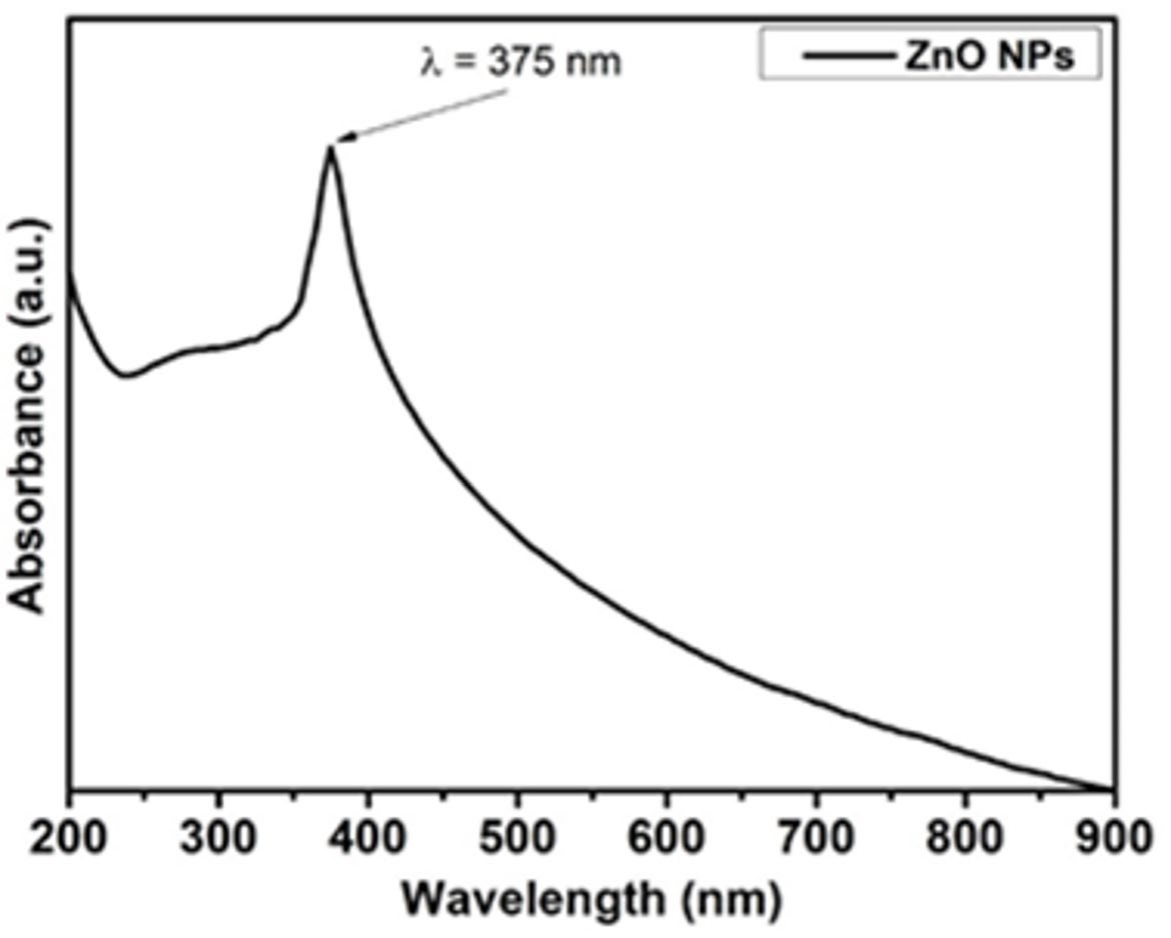
UV-Vis absorption Spectra for ZnO NP.

**Fig. 3:**
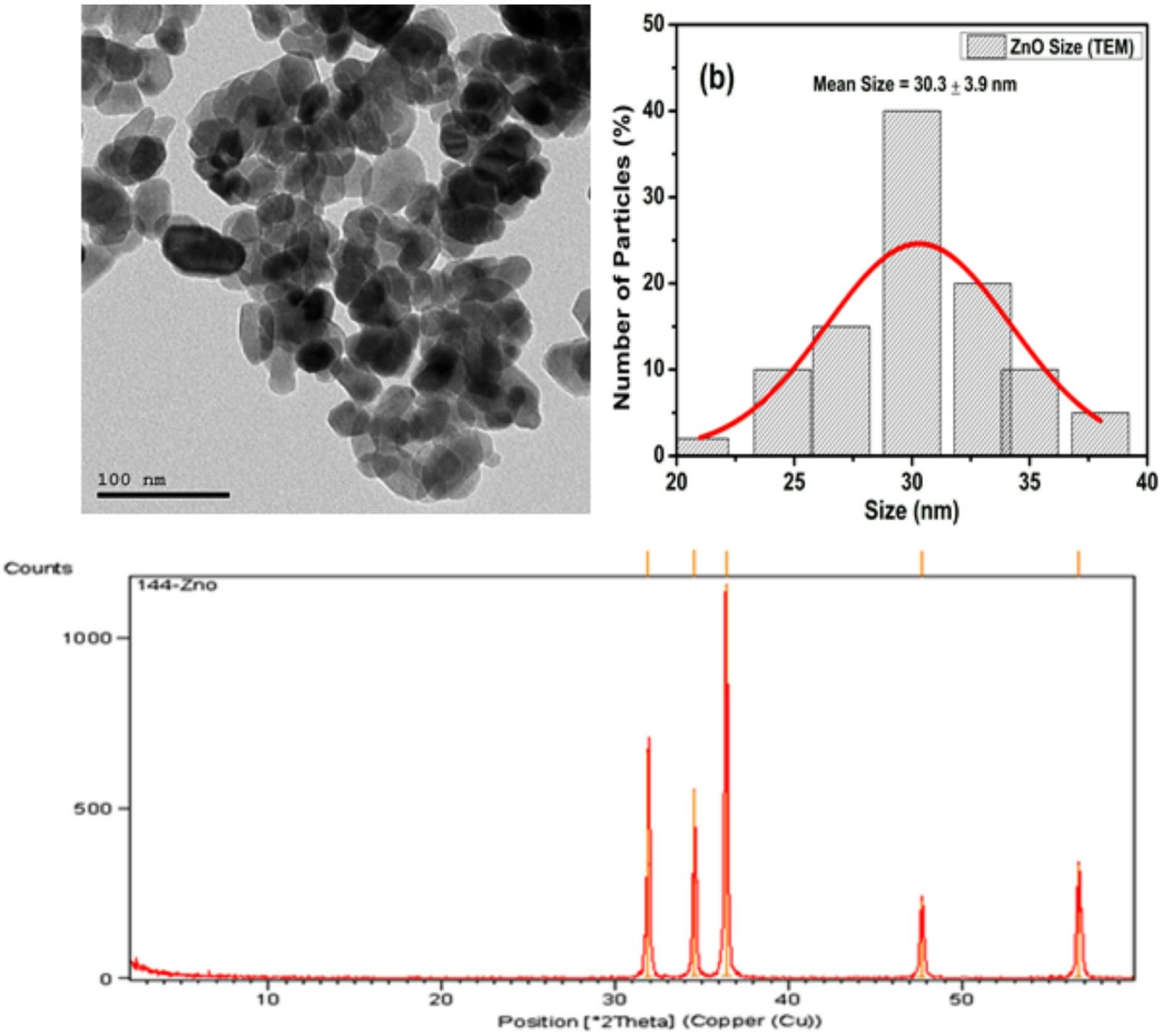
(**a**) TEM images, (**b**) Size distribution and (**c**) XRD patterns of ZnO NPs.

Figure 2 represents the UV–Vis absorption spectra for the as-prepared ZnO NPs. ZnO NPs exhibit a single narrow absorption band at 375 nm ^23^. In addition, the morphological properties of the as-prepared ZnO have been investigated using TEM, as shown in Fig. 3a. In this regards a quasi-spherical (i.e. polyhedral) with an average size ~ 30 ± 3.9 nm as shown in Fig. 3a and b that corresponds ZnO NPs. Furthermore, Fig. 3b illustrated the crystallographic structure for pristine ZnO NPs that investigated using x-ray diffraction (XRD). Five distinct features were obtained at 31.9°, 34.57°, 37°, 47.69°, and 56.75° due to (100), (002), (101), (102), and (110) reflection plans for the hexagonal (i.e., Zincite, ICCD-PDF No. 00-001-1136) crystallographic structure of ZnO NPs, as shown in Fig. 3c.

**Fig. 4:**
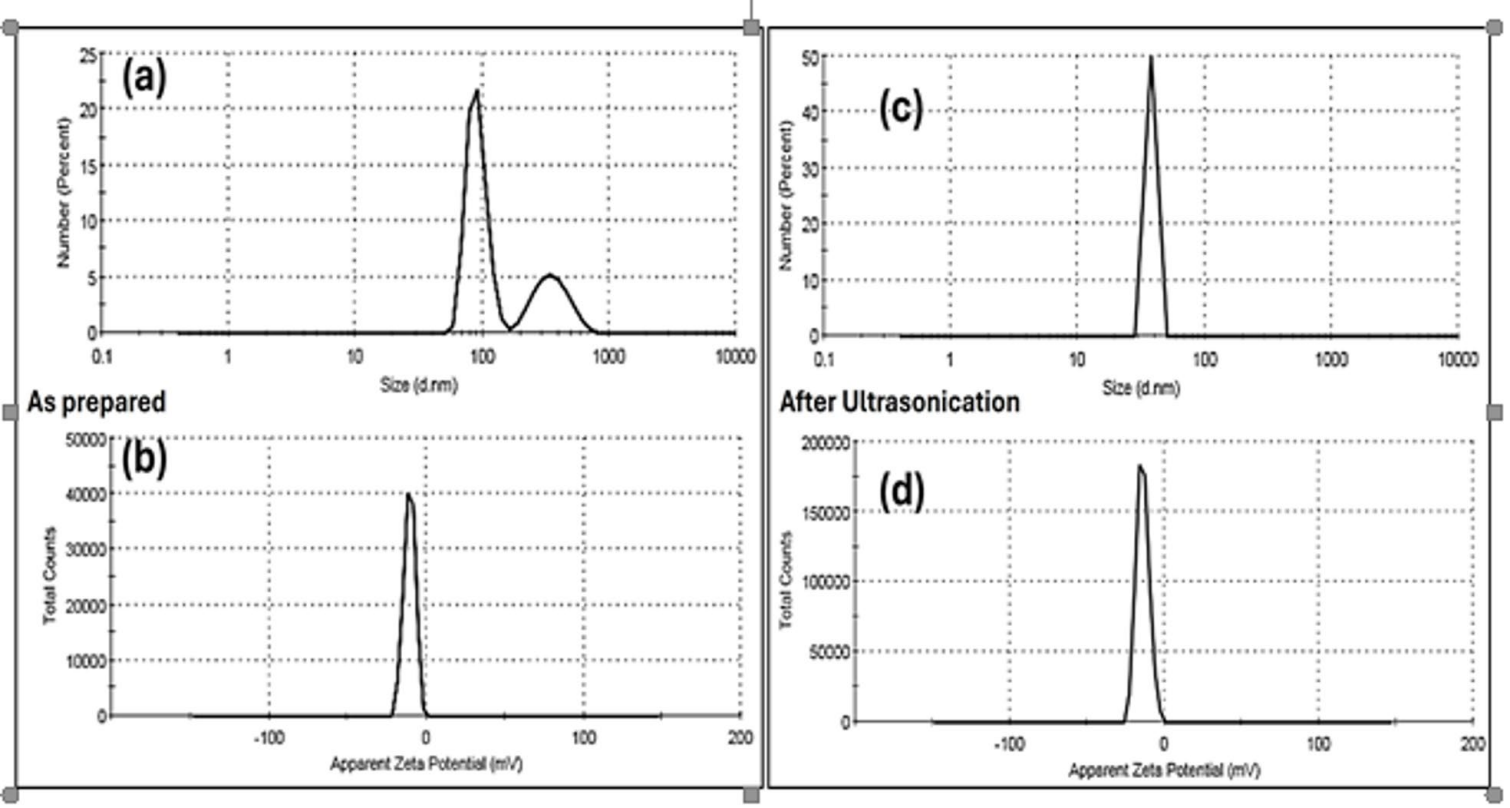
Particle Size distribution using DLS (**a**) as-prepared, and (**c**) after ultrasonication. Zeta potential measurements (**b**) as-prepared and (**d**) after ultrasonication for ZnO NPs.

Finally, colloidal properties including particle size distribution and zeta potential for pristine zinc oxide nanoparticles have been investigated, as shown in Table 2; Fig. 4, respectively. Based on the colloidal properties measurements, the hydrodynamic diameter (H_D_) of as-prepared ZnO NPs was about 91.46 ± 17.44 nm and PDI of 0.988, as shown in Fig. 4a. In addition, the zeta potential (ξ) for ZnO NPs have a negative surface charge with a value of -10.6 mV (See Fig. 4b). Upon the ultrasonication for about 30 min before their application in antimicrobial and anticancer applications, the average particle size was reduced to 59.06 ± 6.129 nm with a reduced PDI of 0.602. In addition, the zetapotential was about − 14, due to better dispersity and lower aggregation after subjection to ultrasonication, as shown in Fig. 4c and d, respectively.

**Table 2:** Colloidal Properties of as-prepared ZnO NPs.

Sample	DLS		Zeta potential (ξ, mV)
	Hydrodynamic Diameter (H_D_, nm)	PDI	
***ZnO*** ***NPs – As prepared***	91.46 ± 17.44	0.982	− 10.6
***ZnO*** ***NPs -High Energy Ultrasonication***	59.06 ± 6.129	0.602	-14

**Fig. 5:**
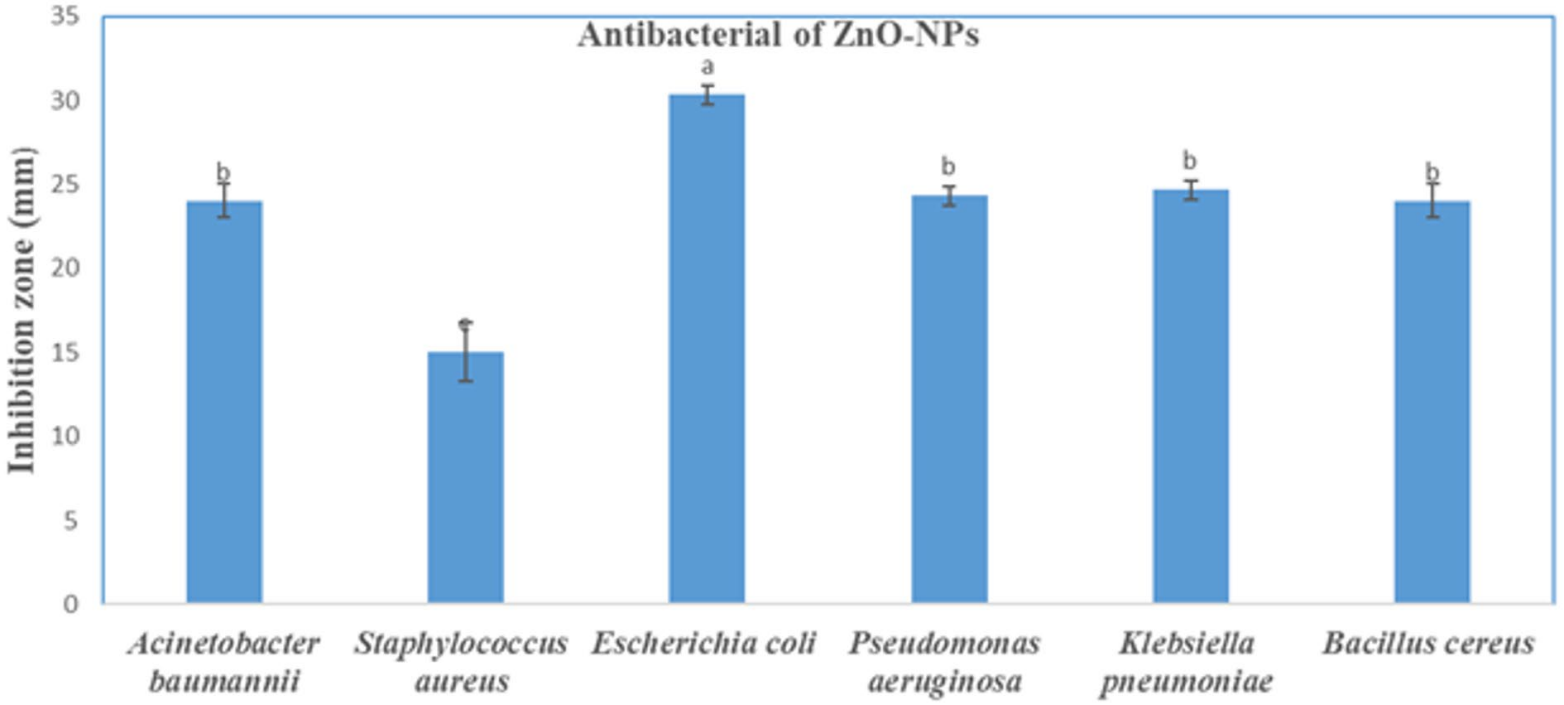
Antibacterial activity of ZnO-NPs, where a, b, and c represented significant letters that calculated from mean of experimental triplicates.

Finally, typical EDX study performed during the imaging of the spheres confirms the presence of Zn and O as shown in Fig. 5. The element mappings show that Zn and O are homogeneously mixed in the spheres. The spectrum shows peaks at 1.5, 8.5, and 9.6 MeV that correspond to Zn, and a peak at 0.9–1.1 MeV which corresponds to O, suggesting that the NPs have a ZnO structure. The line profiles of composition are listed in Table 3.

**Table 3:** EDX Quantitative Analysis of ZnO NPs.

Element	Weight%
O (K)	35.39
Zn (M)	64.61
**Totals**	**100.00**

### Antimicrobial activity

**Fig. 6:**
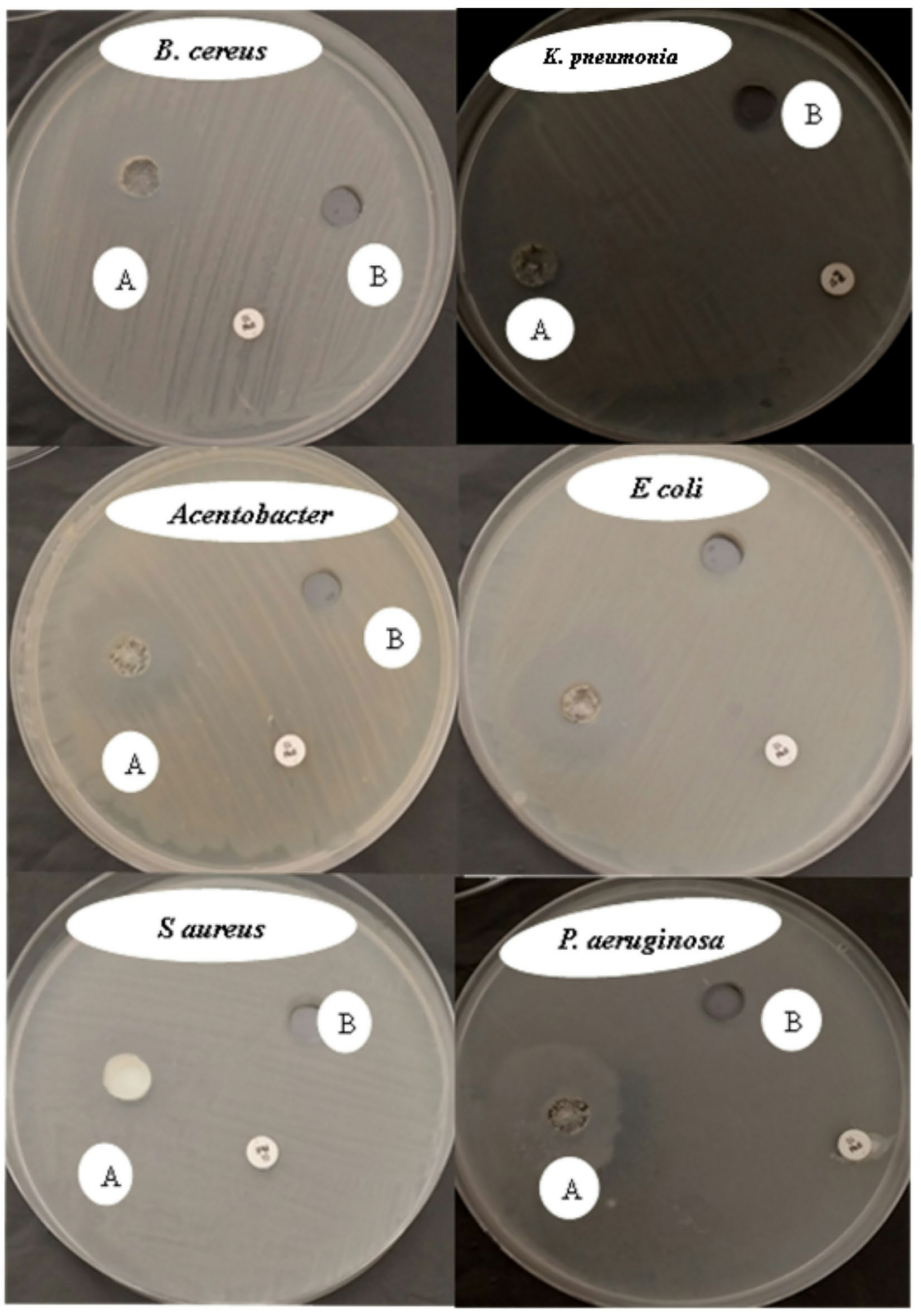
Antibacterial activity of ZnO-NPs on nutrient agar medium, where A- ZnO-NPs, B- Zinc acetate dihydrate (Zn (CH_3_COO)_2_.2H_2_O, 99.99%) as negative control, and antibiotic disc (imipenem 10) as positive control.

Our findings are shown in Figs. 5 and 6 showed that ZnO-NPs (1000 µg/mL) had strong antibacterial activity against all tested bacterial strains. The highest inhibition zone was recorded for *Escherichia coli* (30.33 mm), followed by *Acinetobacter*. (24.66 mm), *Pseudomonas aeruginosa* (24.66 mm), *Klebsiella pneumoniae* (24 mm), *Bacillus cereus* (24 mm), and *Staphylococcus aureus* (15 mm). This suggests that the susceptibility of the bacteria is different for the two types of bacteria. Because ZnO-NPs can produce ROS, damage membrane integrity, and release Zn^2+^ ions, their observed antibacterial activity is consistent with earlier studies showing their efficacy against both Gram-positive and Gram-negative bacteria^24^. *P. aeruginosa* demonstrated decreased susceptibility, most likely as a result of its strong efflux systems and biofilm formation, whereas *K. pneumoniae* demonstrated the highest sensitivity, perhaps as a result of its thinner peptidoglycan layer^25^.

### MIC determination

**Fig. 7:**
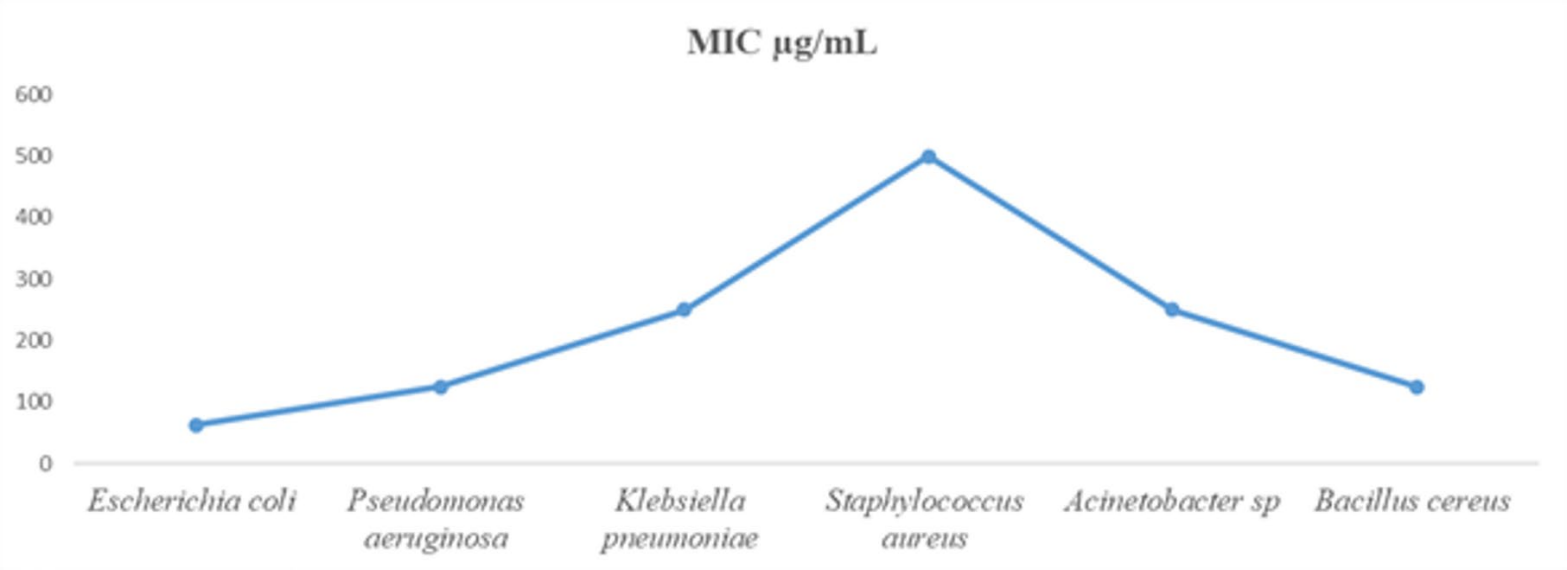
MICs of ZnO-NPs on different isolates.

The MIC values of ZnO-NPs against six clinically significant multidrug-resistant (MDR) bacterial strains are shown in Fig. 7. Each strain’s varying susceptibility is reflected in the wide variations in MIC values (in µg/mL). With a MIC of 500 µg/mL, Staphylococcus aureus has the lowest sensitivity to ZnO-NPs, whereas *Pseudomonas aeruginosa* and *Escherichia coli* have comparatively lower MICs of 62.5 and 125 µg/mL, respectively. *Bacillus cereus* is inhibited at 125 µg/mL, while *Klebsiella pneumoniae* and *Acinetobacter*. need 250 µg/mL for effective inhibition. Differences in bacterial cell wall structure, membrane permeability, efflux mechanisms, and resistance to oxidative stress can be blamed for the observed variation in susceptibility^26^. Due to its thick peptidoglycan layer and effective antioxidant defense mechanisms that neutralize ROS, the main mode of ZnO-NP action, *Staphylococcus aureus* showed the highest MIC (500 µg/mL), indicating lower susceptibility^27^. Because of their thinner cell walls and higher metabolic rates, which worsen ROS-induced damage, *Escherichia coli* and *Pseudomonas aeruginosa*, on the other hand, showed high sensitivity at much lower MICs (62.5 and 125 µg/mL, respectively)^28^. Because of their polysaccharide capsules and capacity to form biofilms, which prevent nanoparticle penetration and ROS diffusion, *Klebsiella pneumoniae* and *Acinetobacter.* have moderate MICs of 250 µg/mL ^29^. These outcomes support earlier research showing that the bactericidal activity of ZnO-NPs is controlled by intricate nano-bio interactions such as particle size, charge, and surface chemistry in addition to concentration^30^.

### Anticancer activity

**Fig. 8:**
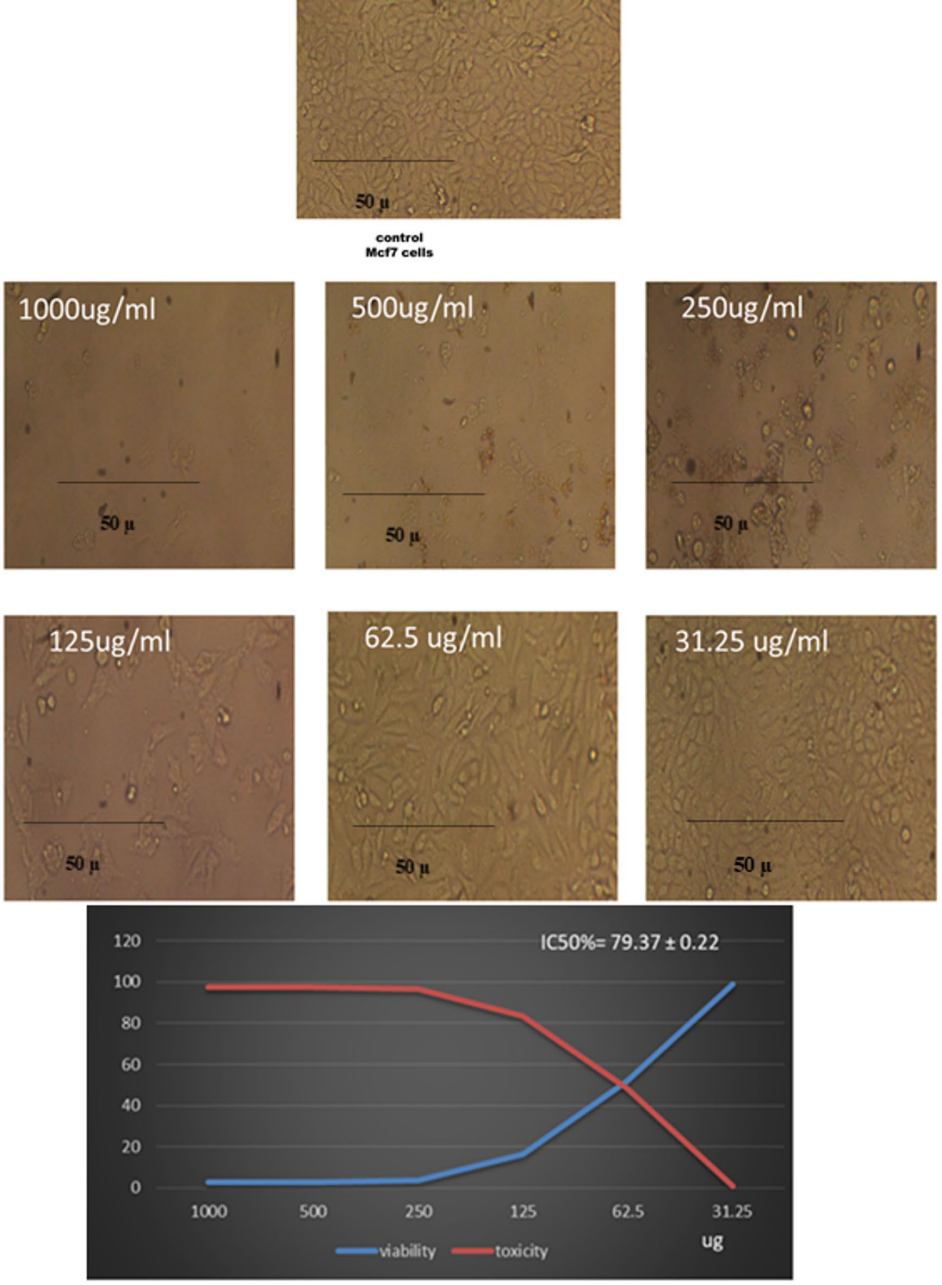
Anticancer activity of ZnO-NPs against Mcf-7, at concentrations 1000 µg/ml, 500 µg/ml, 2500 µg/ml, 125 µg/ml, 62.5 µg/ml, and 31.25 µg/ml.

Our findings are shown in Fig. 8 ZnO-NPs’ cytotoxicity results against the MCF-7 breast cancer cell line demonstrate a definite dose-dependent effect. Cell viability sharply decreased to just 2.47% at the maximum concentration (1000 µg/mL), indicating 97.5% cytotoxicity. With cell viabilities of 2.52% and 3.55%, respectively, concentrations of 500 and 250 µg/mL also demonstrated significant toxicity. At 125 µg/mL, cell viability decreased moderately (16.46%), but at 62.5 µg/mL, it rose to 51.87%, suggesting a cytotoxicity threshold of almost 50%. Viability was almost unaffected (99.15%) at the lowest concentration (31.25 µg/mL), indicating low toxicity. ZnO-NPs successfully inhibit MCF-7 cell proliferation in a concentration-dependent manner, as demonstrated by the calculated IC₅₀ value of 79.37 ± 0.22 µg/mL.

Cell viability significantly decreased from 99.15% at 31.25 µg/mL to only 2.47% at 1000 µg/mL, indicating that the observed cytotoxicity increased with increasing ZnO-NP concentrations. This pattern is in line with earlier research showing that ZnO-NPs mainly cause oxidative stress, interfere with mitochondrial function, and cause cancer cells to undergo apoptosis^31^. The significant reduction in viability at concentrations ≥ 250 µg/mL suggests a potent antiproliferative effect, most likely linked to an increase in intracellular ROS production, which in turn causes DNA damage and apoptosis. Since cancer cells have a higher metabolic rate and a lower antioxidant capacity, ROS-mediated toxicity is generally acknowledged as the main mechanism by which ZnO-NPs produce their anticancer effects^32^. Moderate toxicity (83.5%) was noted at the intermediate concentration of 125 µg/mL, indicating that MCF-7 cells start to approach the apoptotic threshold at this level. ZnO-NPs’ therapeutic potential is supported by the calculated IC₅₀ value of 79.37 ± 0.22 µg/mL, which is within the biologically relevant range and consistent with values reported in the literature for MCF-7 and other human cancer cell lines^33^.

**Fig. 9:**
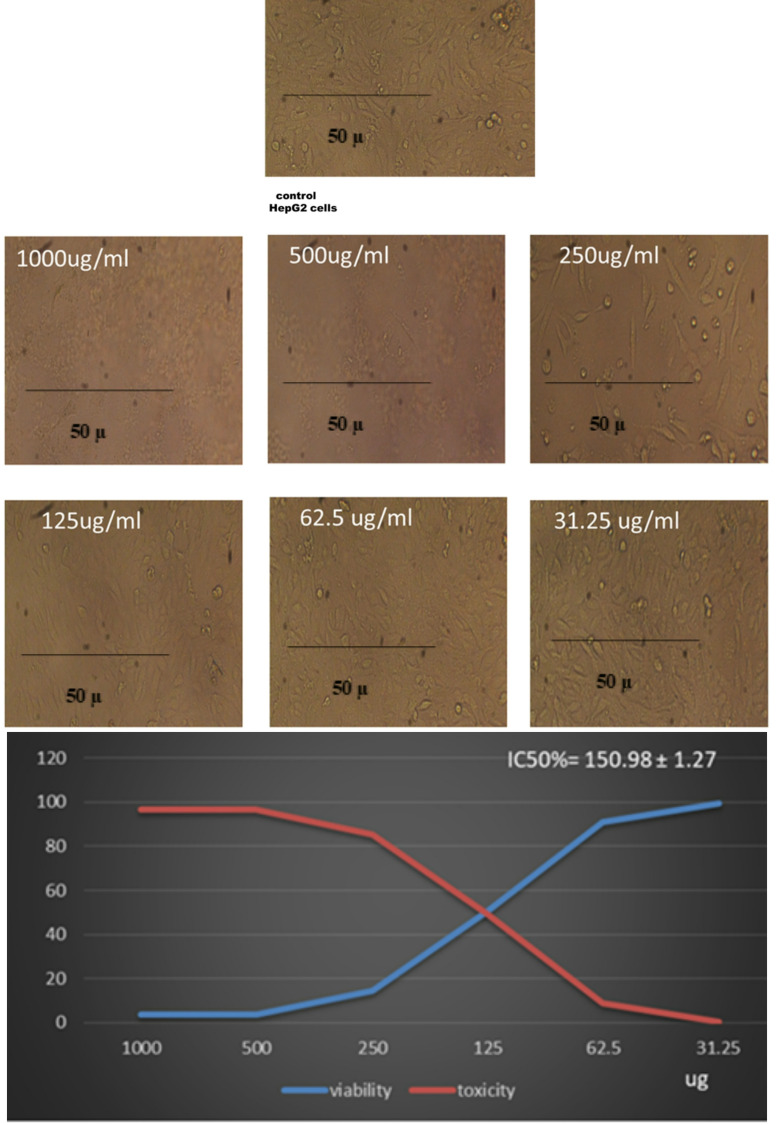
Anticancer activity of ZnO-NPs against HepG2 at concentrations 1000 µg/ml, 500 µg/ml, 2500 µg/ml, 125 µg/ml, 62.5 µg/ml, and 31.25 µg/ml.

The highest concentration of ZnO-NPs, 1000 µg/mL, caused a sharp decline in viability to 3.46%, indicating 96.54% cytotoxicity, according to our results in Fig. 9. Strong anticancer activity at high concentrations was confirmed by the extremely low cell viability (3.41%) at 500 µg/mL. When compared to more sensitive cell lines (e.g., MCF-7, IC₅₀ ≈ 79 µg/mL), this value closely matches the calculated IC₅₀ of 150.98 ± 1.27 µg/mL, which is the concentration needed to reduce viability by 50%. This suggests that ZnO-NPs have a moderate cytotoxic potential against HepG2 cells. Viability was 91% and ~ 99.6% at lower concentrations (62.5 and 31.25 µg/mL), respectively, indicating low cytotoxicity. There was clear damage to the cells, including rounding and shrinkage. Microscopic examination showed noticeable morphological changes at higher treatment dosages, with cells appearing smaller and their membranes becoming more rupturing or disintegrating. These findings point to a therapeutic window during which, at adequate concentrations, ZnO-NPs show selective toxicity toward cancer cells. Consistent with earlier research showing ZnO-NPs cause oxidative stress, mitochondrial dysfunction, and apoptotic cell death in liver cancer cells, our findings imply that ZnO-NPs have potent antiproliferative effects on HepG2 cells at high doses^34^. The computed IC₅₀ of 150.98 ± 1.27 µg/mL indicates that HepG2 cells are moderately sensitive to ZnO-NPs in contrast to other cancer cell lines like MCF-7 (our data shows an IC₅₀ of 79 µg/mL). This is consistent with earlier research that found that the type of cancer cell affects the IC₅₀ values because of differences in membrane permeability, antioxidant capacity, and cellular uptake efficiency^35^.

### Down-regulation of bacterial virulence genes

**Fig. 10:**
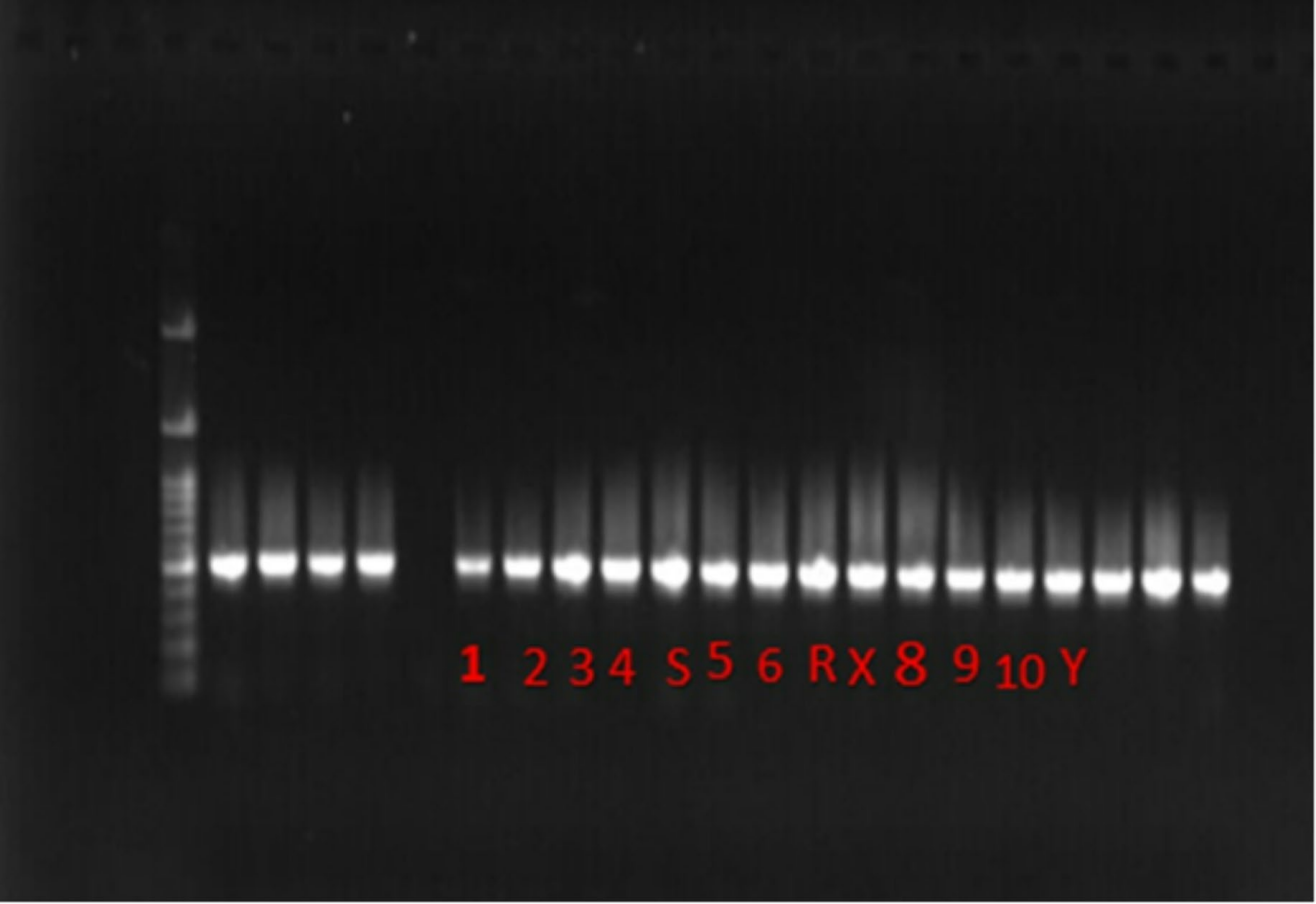
PCR amplification of virulence genes and *recA* as housekeeping gene, where bands 1, and 2 *(luxS)*, 3, and 4 (*rampA*), S negative control for *luxS*, 5, and 6 (fnbA and cna) respectively, R negative control of fnbA, X negative control of *cna*, 8, and 9 (*recA*) sample and positive control respectively from *Staphylococcus aureus* [as example of Gram positive], 10 for (*recA*) positive control of *E.coli* [as example of Gram negative], Y negative control of *recA* of Staphylococcus aureus.

Total RNAs were extracted from bacterial cells that had been prior manipulated with a sublethal concentration of ZnO-NPs (62.5 µg/mL) which lower than both the MIC and MBC of NPs. This was followed by reverse transcriptase reaction, RT-PCR of interest virulence genes, and agarose gel electrophoresis to illustrate detection of PCR products of bacterial virulence genes (Fig. 10).

**Fig. 11:**
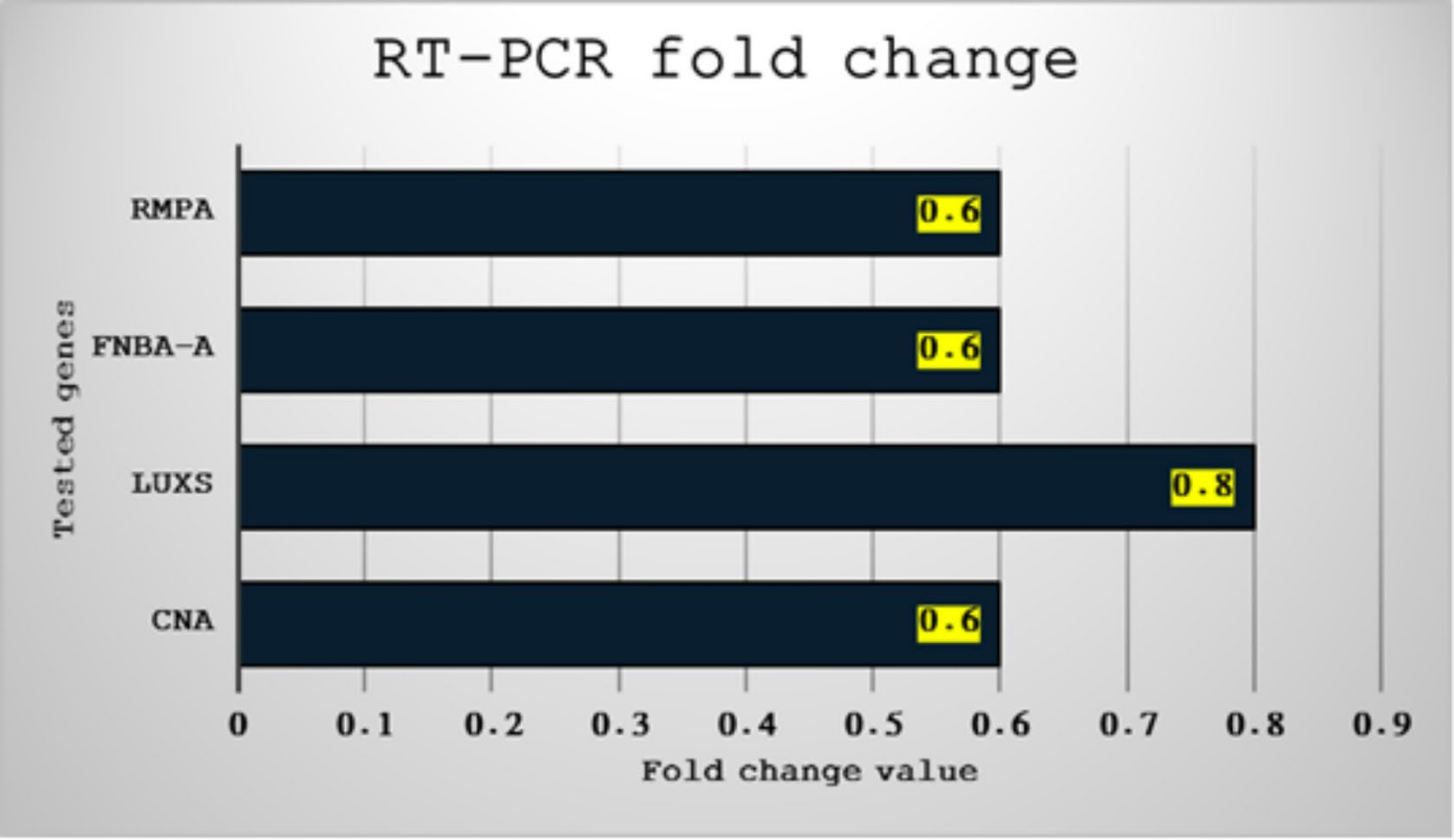
Gene expression fold change of bacterial four virulence factors through qPCR.

Our tested NPs demonstrated a down-regulation effect on virulence factors at concentrations below MIC and MBC on all tested bacterial strains. As shown in Figs. 10 and 11 the most repressed factor was *luxS* (*E. coli*), with a 0.8-fold change, while three genes were down-regulated with a 0.6-fold change: *rmpA* (*K. pneumonia*), *fnbA* (*S. haemolyticus*), and *cna* (*S. aureus*).

Our findings show that even at sub-inhibitory concentrations, or below the MIC, ZnO-NPs significantly downregulate important virulence genes of multidrug-resistant bacteria. ZnO-NPs specifically decreased the expression of *rmpA* in *Klebsiella pneumoniae*, *fnbA* in *Staphylococcus hemolyticus*, and *cna* in *Staphylococcus aureus* by 0.6-fold, signifying a 40% suppression in transcript levels. ZnO-NPs’ capacity to interfere with intercellular communication and coordinated virulence was demonstrated by the notable 0.8-fold repression of *luxS*, a key gene in quorum sensing and biofilm regulation in *Escherichia coli*^36^. These findings are consistent with previous studies showing that metal-based nanoparticles can interfere with bacterial pathogenicity mechanisms by generating ROS and disrupting transcriptional regulation^37^. Our outcomes were corroborated by a study that demonstrated ZnO-NPs’ potent cytotoxicity against MCF-7 cells, causing apoptosis, caspase activation, sub-G1 arrest, and decreased migration^38,39^. The study that found that ZnO-NPs successfully inhibited and disrupted *P. aeruginosa* biofilms, decreased virulence and efflux activity, and restricted host-cell invasion further supported our findings^40^. Our findings were corroborated by a study that found ZnO-NPs had potent antimicrobial and antibiofilm properties against *P. aeruginosa*, inhibiting the quorum-sensing genes, and mature biofilms^41^. Our findings show that ZnO-NPs have potent antivirulence activity even at sub-inhibitory concentrations, indicating that their biological influence extends beyond bactericidal effects. With an average 40% decrease in transcript levels, the nanoparticles dramatically inhibited important virulence genes, such as *rmpA* in *K. pneumoniae*,* fnbA* and *cna* in *S. aureus*. Furthermore, ZnO-NPs’ capacity to disrupt quorum-sensing pathways necessary for biofilm formation and coordinated pathogenicity is demonstrated by the 0.8-fold repression of *luxS* in *E. coli*. These results corroborate earlier research showing that metal-based nanoparticles alter bacterial behavior by producing ROS and interfering with transcriptional regulation, which reduces virulence even in the absence of growth inhibition.

## Conclusion

Our study highlights the strong biological effects of chemically produced ZnO-NPs, showing that they are effective at sub-inhibitory levels in suppressing vital virulence and quorum-sensing genes in addition to preventing bacterial growth and cancer cell proliferation. *rmpA*,* fnbA*,* cna*, and *luxS* have all been shown to be downregulated, which implies that ZnO-NPs can reduce bacterial pathogenicity without encouraging resistance. Furthermore, their potential as a nanotherapeutic agent is supported by their selective cytotoxicity toward cancer cells. Our study promotes the use of ZnO-NPs as a next-generation tool in antimicrobial and anticancer strategies by incorporating gene expression profiling.

## Data Availability

Data is provided within the manuscript.
